# An unusual case of the congenital mesenchymal hamartoma of the neck associated with the midline cervical cleft in neonate

**DOI:** 10.1515/crpm-2021-0094

**Published:** 2022-06-02

**Authors:** Ida Nađ, Dorotea Šijak, Sonja Anić Jurica, Antonia Jakovčević

**Affiliations:** Department of Neonatology at Department of Gynaecology and Obstetrics, University Hospital Centre Zagreb, Zagreb, Croatia; Department of Pediatrics, University Hospital Centre Zagreb, Zagreb, Croatia; Department of Pathology and Cytology, University Hospital Centre Zagreb, Zagreb, Croatia

**Keywords:** congenital mesenchymal hamartoma, median cervical cleft, neck contracture, neonate, pediatric neck mass

## Abstract

**Objectives:**

Hamartomas are non-neoplastic developmental anomalies, mostly congenital, characterized by uncontrolled, disorganized proliferation of local endogenous tissue, which can normally be found at the site of origin and are very often mesodermally derived. It is well known that hamartoma can be associated with congenital midline cervical cleft and therefore cause a variety of symptoms. In general, they are benign and indolent, but they can be the cause of complex morbidity if they are localized within specific regions, such as the head and neck, which represent highly sensitive and vulnerable areas.

**Case presentation:**

The reported case is unusual because of the presence of a congenital mesenchymal hamartoma along with the median cervical cleft, in a 1-day-old neonate, without the presence of any respiratory symptoms or associated congenital features. Although extremely rare, hamartomas should be included in the differential diagnosis of congenital neck masses, with emphasis on diagnostic approach, to avoid overly aggressive treatment and possible complications, such as infection, further mass growth, malignant transformation and compression of the adjacent neck structures.

**Conclusions:**

Appropriate and timely treatment of the hamartoma of the neck in neonates, with further follow-up is necessary to avoid an overly aggressive treatment and to distinguish benign from malignant lesions, which is necessary for successful curative outcome.

## Introduction

The entity “hamartoma” was first established by Albrecht in 1904, as a term which explained and emphasized the difference between tumor-like, benign lesions and true neoplasms [1]. Hamartomas are described as non-neoplastic developmental anomalies, mostly present from birth, characterized by uncontrolled and disorganized proliferation of local endogenous tissue elements, which can normally be found at the site of origin and are very often mesodermally derived, but they can also develop from any of the three germinal layers [2]. In most of the cases, their growth is limited and follows the general development of the whole body, they are mostly asymptomatic and occur isolated in healthy individuals [1].

The multiple presentations of solitary hamartomas are *PTEN* (phosphatase and tensin homolog) hamartoma tumor syndromes, such as *Cowden* syndrome and *Proteus* syndrome, or less commonly as a part of tuberous sclerosis [3], [4], [5], [6]. Pathological sub-classification of hamartomas depends on the type of predominant endogenous tissue and its variations, so we can distinguish: vascular, muscle predominant, adipose tissue predominant and intramuscular capillary variants, where a combination of mature capillaries which are localized between and around muscle bundles is present [7].

In general, they are benign, indolent, but they can be the cause of certain complex morbidity, with their inclusion and spreading inside specific vulnerable regions, particularly the head and neck, which represent highly sensitive areas [8].

We report the case of a neonate with a congenital median cervical cleft and associated cervical mass which turned out to be a mesenchymal hamartoma covered by respiratory epithelium.

## Case presentation

We are presenting a full-term vital male newborn, born vaginally, with a gestational age of 38 + 6 weeks, birth weight (BW) 3,260 g, birth length (BL) 48 cm, AS 10/10, which has been referred to our Department of Neonatology for further evaluation of a midline cervical cleft along with an unidentified neck mass of unknown origin. The course of the pregnancy was complicated, with hyperstimulation syndrome as part of the IVF (*in vitro* fertilization) procedure which included using progesterone and myo-inositol among others (9th day after embryo transfer), but was otherwise regular.

Initially, an obvious skin defect was noticed at birth as a suspected mucosal protrusion at the anterior part of the neck, with possible secretion (about 2 cm in long diameter). The described mass was firm, arising from the median cervical cleft, well separated from surrounding neck regions, without visible communication with parotid or salivary glands. At first, possible differential diagnosis included thyroglossal duct cyst, dermoid and branchial cyst, as well as some malformation of gill arches and protrusion of esophageal mucosa. Otherwise, no abnormality in physical appearance has been detected.

### Diagnosis and surgical management

**Figure 1:**
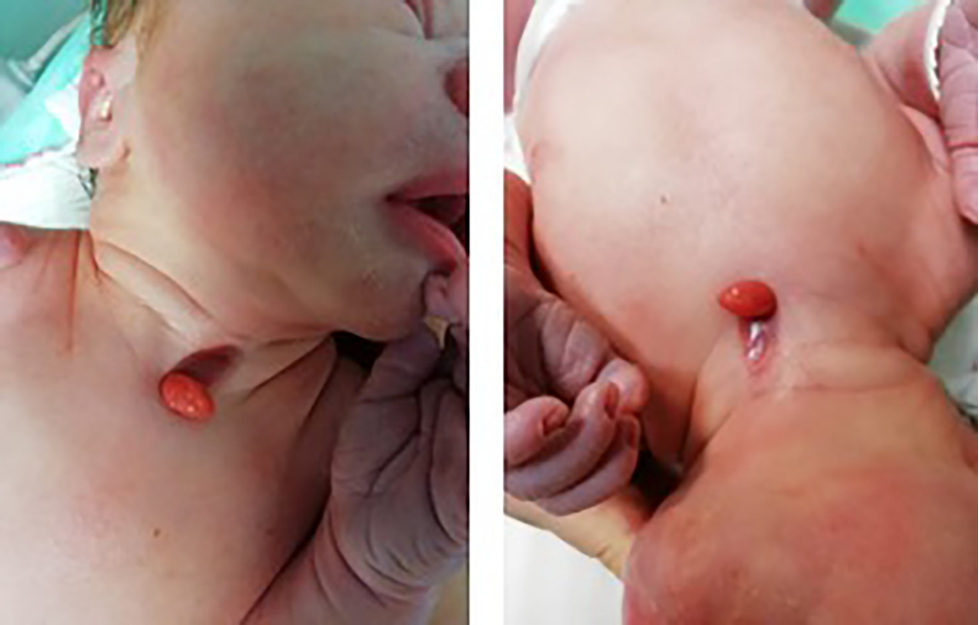
Congenital midline cervical cleft (CMCC) from the upper side-midline skin defect with protruded oval shaped neck mass (lump) of unknown origin.

Otorhinolaryngologic examination demonstrated a visible tumor formation in the median line of the neck on a relatively narrow base, about 2.5 × 0.5 cm in size, moderately hard on palpation with altered skin area around the formation of an oval shape. Below the described formation, there was a smaller skin area that looked more like a mucous membrane, but without visible fistula (Figure 1). First it was described as a possible anomaly of gill arches or a thyroglossal anomaly, because of the specific median position.

Ultrasound examination of the neck regions did not reveal pathologically altered lymph nodes. Both parotid and submandibular glands as well as the thyroid gland had regular dimensions and echogenicity, with a homogeneous structure without focal lesions. There was no dilation of the main salivary ducts. In the median line of the neck, referred to as a VI neck region (radiological region IV), there was a visible hypoechogenic skin area, with clearly limited formation of dimensions 1.2 × 1.2 × 0.9 cm without communication with the thyroid gland. Between the formation and the thyroid gland there was a small lymph node with a diameter of 0.3 cm. The formation showed no enhanced vascularization. There was no clear communication with the thyroid gland in terms of open thyroglossal ductus. The thymus was localized mediastinal with typical echogenicity without visible ectopic localizations. Other posterior neck structures have been described as within physiological limits in all regions.

Surgical treatment included excision of the exophytic formation at the front of the neck in the median line. During the procedure the sinus has been shown, with a depth of 3 mm (measured with the test). No communication with other posterior structures of the neck was demonstrated.

In the further clinical course, at the age of 1 year and 6 months, a re-evaluation was performed, which showed normal neck mobility, without functional limitations and the absence of any signs of respiratory or digestive difficulties, such as dyspnoea, respiratory distress, upper respiratory infections, feeding problems or persistent communication of neck structures and the digestive tract.

The purpose of the second surgical act was to improve the functional and cosmetic appearance of the scar, by using of Z-plasty technique which would redirect scar tissue into better alignment within natural skin folds and lines of least skin tension, which may lengthen the already contracted scars. This was done 1 year and 8 months after the primary surgical treatment. The postoperative course passed without complications with adequate wound healing. An additional tissue sample has been taken for pathohistological analysis, which confirmed, along with the scar and adipose tissue in the dermis layer, a focal tubular formation lined with respiratory epithelium and surrounded by abundant lymphoid tissue, which was equivalent to the initial PHD findings.

Long-term follow-up has been continued by otorhinolaryngologists, pediatricians and dermatologists, every 3–6 months. There was a stable clinical course, without associated complications and satisfactory cosmetic appearance of the scar, as well as full functioning of the affected region. Further follow-up is necessary because of the potential development of keloids, contractures, malignant transformation which can result with secondary respiratory and digestive problems.

### Pathological findings

**Figure 2:**
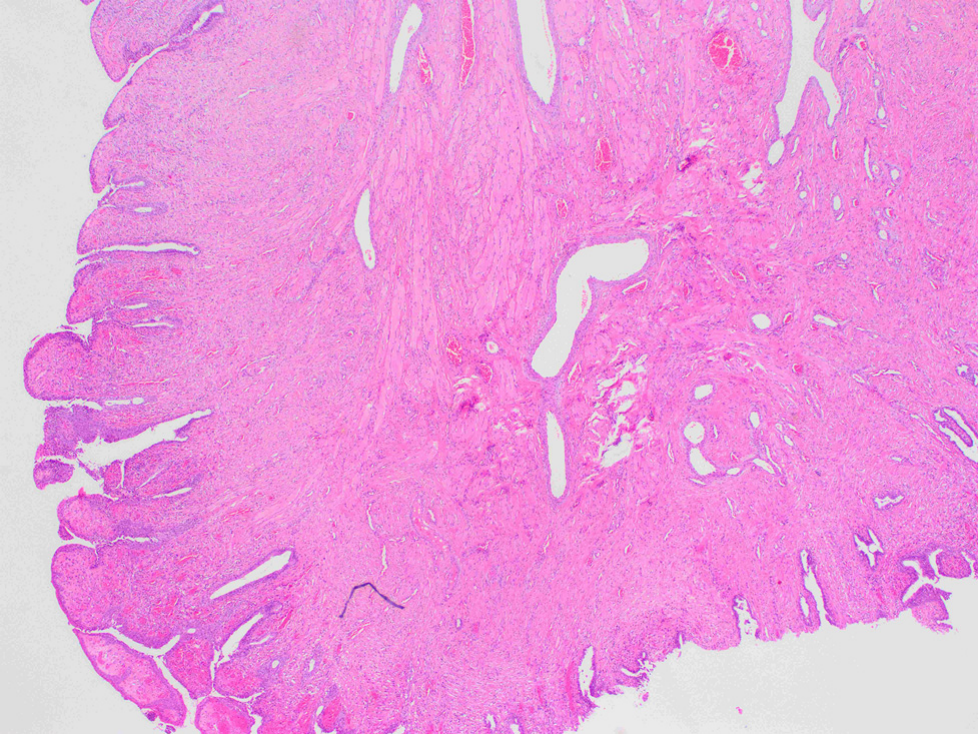
Histology of the specimen. A polypus covered by layer of columnar epithelium with visible skeletal muscle tissue and dilated blood vessels in the middle (magnification ×2, hematoxylin–eosine staining).

The observed material is a polyposis formation of 1.8 × 1 cm in size, on a peduncle of 0.4 cm in diameter. Histologically, it consists of skeletal and smooth muscle tissue fibers, individual seromucous glands, circulatory tracts, branches of the peripheral nerves and connective tissue, while on the surface, which is partially villous and eroded, there is a ciliated, pseudostratified columnar epithelium with areas of squamous epithelial metaplasia. The subepithelial layer is composed of stroma which consists of multiple glands lined with the typical ciliated, columnar epithelium (Figures 2 [Fig j_crpm-2021-0094_fig_003]–4).

**Figure 3: j_crpm-2021-0094_fig_003:**
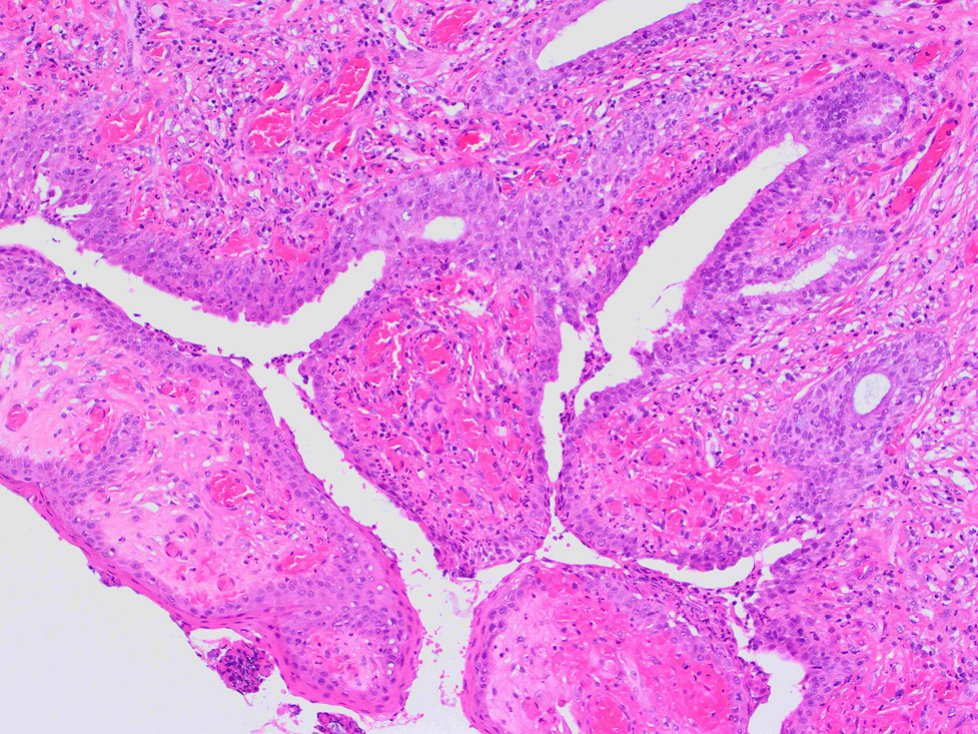
Histology of the specimen. Surface of mesenchymal hamartoma covered with columnar epithelium and partially metaplastic squamous epithelium (magnification ×10, hematoxylin–eosine staining).

**Figure 4:**
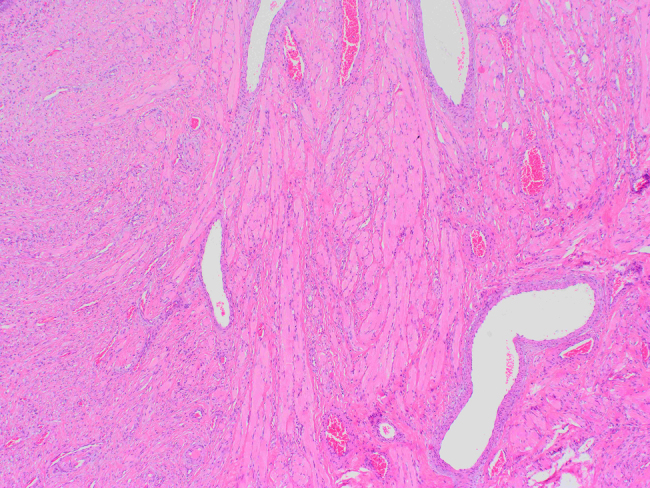
Histology of the specimen. Structure of skeletal muscle tissue (magnification ×4, hematoxylin–eosine staining).

Finally, the described histological changes confirmed a mesenchymal hamartoma and are consistent with initial clinical diagnosis of the congenital midline cervical cleft (CMCC).

## Discussion

Mesenchymal hamartomas of the head and neck usually involve the tongue, followed by the palate and alveolus, pharynx, larynx and sinonasal cavity [7], [8], [9]. It is well known that CMCC can be associated with related cervical masses, such as hamartoma. At birth, the external layer of the cleft may consist of a weeping, red membrane which then heals to produce cicatricial skin as the patient grows. The fibrous cord, which usually extends down to the pretracheal fascia, similar to this case, also becomes more prominent as the child grows. This is because the affected tissues lag behind in vertical growth compared to the surrounding normal neck tissue [10].

Congenital midline cervical cleft may be associated with other embryologic defects, such as median mandibular and sternal clefts, anomalies of thyroid cartilages thyroglossal duct cysts, bronchogenic cysts, and cardiac anomalies [10].

In general, clinical, pathological and imaging characteristics are crucial for differentiation of the neck masses. Most of the lesions in children have an origin which is either congenital or inflammatory and the neck is an uncommon localization for them. The most common congenital developmental masses in the neck are thyroglossal duct cysts, branchial cysts, dermoid cysts as well as vascular malformations (such as capillary and cavernous hemangiomas) [7].

The differential diagnosis of the head and neck mass in the pediatric patient is wide. More common benign neoplastic lesions include hemangiomas and vascular malformations, neurofibromas, choristomas (dermoid cysts and teratomas), and parotid neoplasms. Malignant lesions include rhabdomyosarcoma, lymphoma, and parotid cancer. Infections and inflammatory lesions must also be considered [8].

We report the unusual case of mesenchymal hamartoma with elements of respiratory epithelium protruding from the midline cervical cleft in neonate. Considering the unknown origin of hamartoma and the possible existence of fistula, ultrasound and oral ingestion of methylene blue was performed for detailed preoperative planning and complete surgical excision, as well as for exclusion of possible communication with other neck structures. The analysed specimen revealed no evidence of malignant transformation. The final surgical management of the median cervical cleft has been delayed until the child reaches the age of 1 year to reduce the possibility of developing postoperative neck contracture, as recommended in recent literature [10].

Possible clinical adverse outcomes include two major groups of pathology, those of respiratory tract such as dyspnoea due to the specific localization and size of hamartoma, upper respiratory infection and respiratory distress, and those of digestive tract like feeding problems as a result of difficulty swallowing, or presence of communication between the digestive tract and skin (sinus) or fistula, which can be accompanied by recurrent infections, inflammation of surrounding skin, tissue maceration and which may be associated with mandibular and sternal cleft.

In conclusion, referring to the paucity of the cases, this is an example of unusual congenital mesenchymal hamartoma of the neck associated with midline cervical cleft without respiratory or digestive symptoms, where a detailed diagnostic and therapeutic approach was crucial for individual patient treatment, which includes avoiding an overly aggressive treatment of this benign lesion and further long-term follow-up for complete curative outcome.
